# Depletion of Rictor, an essential protein component of mTORC2, decreases male lifespan

**DOI:** 10.1111/acel.12256

**Published:** 2014-07-25

**Authors:** Dudley W Lamming, Maria M Mihaylova, Pekka Katajisto, Emma L Baar, Omer H Yilmaz, Amanda Hutchins, Yetis Gultekin, Rachel Gaither, David M Sabatini

**Affiliations:** 1 Department of Medicine, University of WisconsinMadison, WI, 53705, USA; 2 William S. Middleton Memorial Veterans HospitalMadison, WI, 53705, USA; 3 Whitehead Institute for Biomedical ResearchCambridge, MA, 02142, USA; 4 Department of Biology, MITCambridge, MA, 02139, USA; 5 Howard Hughes Medical Institute, MITCambridge, MA, 02139, USA; 6 Broad Institute of Harvard and MIT, Seven Cambridge CenterCambridge, MA, 02142, USA; 7 The David H. Koch Institute for Integrative Cancer Research at MITCambridge, MA, 02139, USA; * Institute of Biotechnology, University of HelsinkiHelsinki, Finland; † Salk Institute for Biological StudiesSan Diego, CA, 92037, USA; ‡ The Rockefeller UniversityNew York, NY, 10065, USA

**Keywords:** aging, gender dimorphism, longevity, mTORC2, Rictor, Rapamycin

## Abstract

Rapamycin, an inhibitor of the mechanistic target of rapamycin (mTOR), robustly extends the lifespan of model organisms including mice. We recently found that chronic treatment with rapamycin not only inhibits mTOR complex 1 (mTORC1), the canonical target of rapamycin, but also inhibits mTOR complex 2 (mTORC2) *in vivo*. While genetic evidence strongly suggests that inhibition of mTORC1 is sufficient to promote longevity, the impact of mTORC2 inhibition on mammalian longevity has not been assessed. RICTOR is a protein component of mTORC2 that is essential for its activity. We examined three different mouse models of *Rictor* loss: mice heterozygous for *Rictor*, mice lacking hepatic *Rictor*, and mice in which *Rictor* was inducibly deleted throughout the body in adult animals. Surprisingly, we find that depletion of RICTOR significantly decreases male, but not female, lifespan. While the mechanism by which RICTOR loss impairs male survival remains obscure, we find that the effect of RICTOR depletion on lifespan is independent of the role of hepatic mTORC2 in promoting glucose tolerance. Our results suggest that inhibition of mTORC2 signaling is detrimental to males, which may explain in part why interventions that decrease mTOR signaling show greater efficacy in females.

## Introduction

mTOR (mechanistic Target of Rapamycin) is a protein kinase that functions as a central mediator of growth and metabolism in response to environmental stimuli (Laplante & Sabatini, 2012). mTOR is found in two different complexes with distinct substrates. mTOR complex 1 (mTORC1) is sensitive to the availability of amino acids and glucose, and promotes growth and mRNA translation through numerous substrates that include S6K1, 4E-BP1, and ULK1 (Kang *et al*., 2013). mTOR complex 2 (mTORC2) is regulated by growth factor signaling and regulates a diverse set of substrates, including specific regulatory residues of AKT, serum/glucocorticoid regulated kinase (SGK), and protein kinase C α (PKCα).

Rapamycin, a small molecule that is FDA-approved as an immunosuppressant and for the treatment of certain cancers, promotes longevity in yeast, worms, flies, and in mice (Medvedik *et al*., 2007; Bjedov *et al*., 2010; Miller *et al*., 2011; Robida-Stubbs *et al*., 2012). Rapamycin is an acute inhibitor of the mechanistic target of rapamycin (mTOR) complex 1, and experiments in model organisms up to and including mice demonstrate that inhibition of mTORC1 signaling is sufficient to extend lifespan (Selman *et al*., 2009; Lamming *et al*., 2012). Intriguingly, female mice treated with rapamycin show a greater extension of lifespan than do male mice, a trait shared by mice lacking the mTORC1 substrate *S6K1* (Selman *et al*., 2009; Miller *et al*., 2013; Zhang *et al*., 2013).

We have demonstrated that chronic, long-term administration of rapamycin results in inhibition of not only mTORC1, but also mTORC2, *in vitro* in cancer cell lines as well as *in vivo* in mice (Sarbassov *et al*., 2006; Lamming *et al*., 2012). Long-term rapamycin treatment results in glucose intolerance in humans, rats, and C57BL/6 mice as well as genetically heterogeneous HET3 mice (Houde *et al*., 2010; Gyurus *et al*., 2011; McCormack *et al*., 2011; Lamming *et al*., 2013). We found that this effect is mediated in part by decreased hepatic insulin sensitivity, an effect that can be reproduced by depletion of RICTOR, an essential protein component of mTORC2, in the whole body (Lamming *et al*., 2012) or specifically in the liver (Hagiwara *et al*., 2012; Lamming *et al*., 2012; Yuan *et al*., 2012). We have recently shown that *Rictor* deletion specifically in the liver has significant effects at both the mRNA and phosphoproteomic level that are distinct from those seen with acute rapamycin treatment (Lamming *et al*., 2014).

Although mice treated with rapamycin have a significant increase in lifespan, the effect of decreased mTORC2 signaling on mammalian longevity is unclear. In *C. elegans*, RNAi against *Rictor* in adult worms results in a significant increase in lifespan (Robida-Stubbs *et al*., 2012). In contrast, a *Rictor*-null mutant strain of *C. elegans* has decreased longevity on some diets (Soukas *et al*., 2009). The impact of mTORC2 disruption on mammalian longevity is unknown; understanding the consequences of this inhibition will significantly inform the development of mTOR inhibitors as potential therapies for age-related diseases.

To elucidate the role of mTORC2 in mammalian lifespan, we used three different mouse models of RICTOR depletion: mice heterozygous for *Rictor*, mice in which *Rictor* was deleted specifically in the liver, and mice in which *Rictor* was inducibly deleted in an adult mouse through the use of a ubiquitously expressed tamoxifen-responsive Cre recombinase. We find that depletion of RICTOR significantly decreases male, but not female, lifespan. Both male and female mice lacking hepatic *Rictor* showed decreased glucose tolerance, while both male and female mice heterozygous for *Rictor* have normal glucose tolerance, suggesting the male-specific decrease in lifespan does not result from decreased glucose tolerance or insulin resistance.

## Results

### Depletion of RICTOR impairs male, but not female, longevity

**Figure 1:**
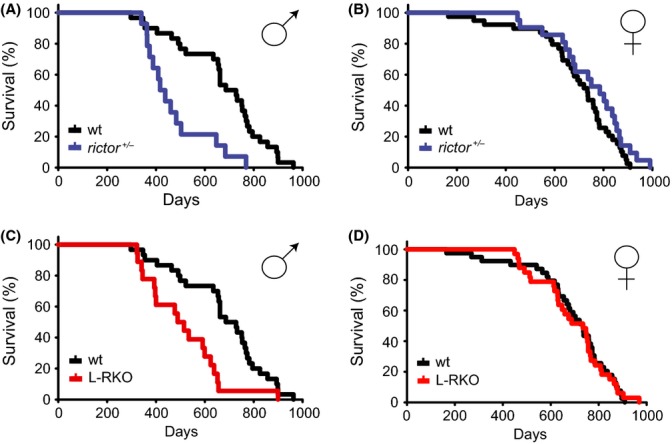
Depletion of RICTOR impairs male, but not female, lifespan. (A,B) Kaplan–Meier plots showing lifespans of (A) male and (B) female mice heterozygous for *Rictor*. (C,D) Kaplan–Meier plots showing lifespans of (C) male and (D) female mice in which *Rictor* was deleted specifically in the liver (L-RKO). Littermate control mice (wt) of *rictor*^*+/−*^ and L-RKO mice were pooled for analysis, and control lifespan curves are duplicated in A/C and B/D. The survival of 155 mice was analyzed as follows: Females (39 wild-type, 21 *rictor*^*+/−*^, 33 L-RKO), Males (30 wild-type, 14 *rictor*^*+/−*^, 18 L-RKO). Raw data and statistical information can be found in Tables S1 and S2.

To determine the role of mTORC2 signaling in mammalian lifespan, we first examined two different mouse models with decreased expression of RICTOR, an essential protein component of mTORC2. We examined the lifespan of male and female mice that were either heterozygous for *Rictor* (*rictor*^*+/−*^) or in which *Rictor* was deleted specifically in the liver (L-RKO); the pooled wild-type littermates of both *rictor*^*+/−*^ and L-RKO mice were used as controls. Male *rictor*^*+/−*^ mice had a significantly decreased lifespan, with a 40% decrease in median lifespan compared to wild-type controls (Fig. 1A, Table S1 and S2 in Supporting Information). In contrast, female *rictor*^*+/−*^ mice had a lifespan that was indistinguishable from wild-type (Fig. 1B). We observed a similar effect in L-RKO mice, with a 30% decrease in median lifespan in male L-RKO mice, while female L-RKO survival was indistinguishable from wild-type (Fig. 1C, 1D). Aged male *rictor*^*+/−*^ and L-RKO mice were indistinguishable from wild-type controls and had equivalent rotarod performance to age-matched control mice (Figure S1 in Supporting Information). There was a significant decrease in the incidence of cancer observed at death in *rictor*^*+/−*^ and L-RKO male mice compared to wild-type controls, likely due to their death prior to the onset of cancer; there was no statistically significant effect of genotype on cancer in females (Table S3).

**Figure 2:**
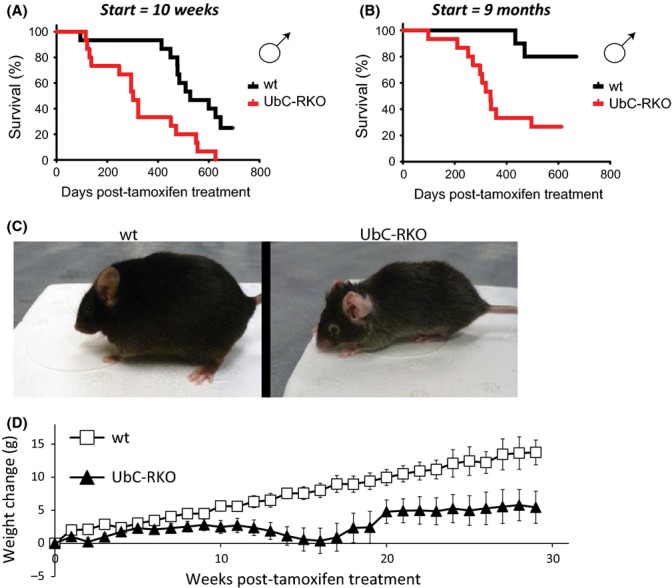
Inducible depletion of RICTOR impairs male lifespan. (A) Kaplan–Meier plot showing survival of male wild-type and UbC-RKO mice treated with tamoxifen at 10 weeks of age. The survival of 26 mice was analyzed as follows: Males (11 wild-type, 15 UbC-RKO). (B) Kaplan–Meier plot showing survival of male wild-type and UbC-RKO mice treated with tamoxifen at 9 months of age. The survival of 25 mice was analyzed as follows: Males (10 wild-type, 15 UbC-RKO). Raw data and statistical information can be found in Tables S1 and S2. (C) Depletion of RICTOR results in gray hair and kyphosis. Pictured: 15-month-old wild-type (left) and UbC-RKO mice (right) from which *Rictor* was excised at 10 weeks of age. (D) Weight change of wild-type and UbC-RKO mice over 30 weeks, starting at 10 weeks of age.

These first two mammalian models of RICTOR depletion we studied lacked normal expression of RICTOR starting early in life. To address the possibility that RICTOR depletion starting later in life would prove more beneficial to males than constitutive depletion, we bred mice with a conditional allele of *Rictor* and a ubiquitously expressed tamoxifen-inducible Cre recombinase (UbC-RKO). We treated UbC-RKO mice with tamoxifen for 1 week at 10 weeks of age (Fig. 2A), and observed a significant decrease in lifespan; indeed, mice with *Rictor* deleted at 10 weeks of age had a median survival of less than a year (Table S1). Many studies of rapamycin have commenced treatment at 9 months of age or later. To explore the possibility that deletion of *Rictor* later in life would prove beneficial, we aged control and UbC-RKO mice to 9 months of age, and then treated these mice with tamoxifen for 1 week. We found that depletion of RICTOR significantly impaired survival even when begun late in life (Fig. 2B).

We were unable to determine cause of death of male UbC-RKO mice; however, only a single mouse with *Rictor* deleted at 10 weeks of age was found to have cancer upon necropsy, suggesting that cancer was not the cause of death. We observed a number of interesting phenotypes in UbC-RKO male mice treated with tamoxifen, including the development of gray hair, hunching, and dermatitis around the neck, with inflammation of the pinna (Fig. 2C). UbC-RKO male mice were also significantly lighter than their wild-type littermates, gaining less weight following treatment with tamoxifen (Fig. 2D). Although we were unable to conduct a formal lifespan study of female UbC-RKO mice, UbC-RKO mice treated with tamoxifen at 10 weeks of age to excise *Rictor* appeared phenotypically normal, and we did not observe premature death in these mice.

In parallel with the UbC-RKO lifespan study performed starting at 10 weeks of age shown in Figure 2(A), in which mice were fed *ad libitum* (AL), we placed a cohort of wild-type and UbC-RKO mice on a calorie-restricted (CR; 60% of AL food intake) diet starting at 12 weeks of age. We found that a CR diet did not rescue the short lifespan of the UbC-RKO mice (Figure S2A), despite UbC-RKO mice achieving a similar weight loss compared to wild-type mice on a CR diet (Figure S2B). UbC-RKO mice on a CR diet had a trend toward improved rotarod performance and glucose tolerance, but did not benefit as much as wild-type mice on a CR diet (Figure S2C, S2D). UbC-RKO mice on a CR diet had a trend toward improved rotarod performance and glucose tolerance, but did not reach the same high level of physical performance that wild-type mice on a CR diet do during a rotarod test; similarly, wild-type mice on a CR diet had a lower area under the curve during a glucose tolerance test than UbC-RKO mice on a CR diet (Figure S2C, S2D).

### Decreased phosphorylation of AKT T450 and S473 in both male and female L-RKO mice

**Figure 3:**
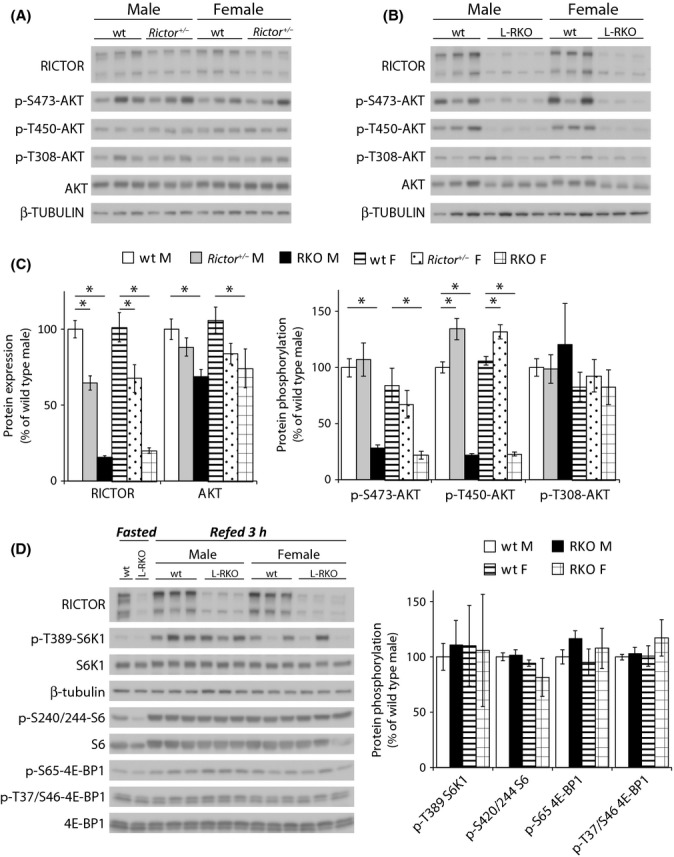
Analysis of mTOR signaling in the livers of L-RKO and *rictor*^*+/−*^ mice. (A,B) Analysis of protein expression and phosphorylation in the livers of wild-type and (A) *rictor*^*+/−*^ and (B) L-RKO mice fasted overnight and then refed for 3 h. (C) Quantification of the expression of RICTOR and AKT relative to β-tubulin, and the phosphorylation of AKT S473, T450, and T308 relative to AKT. Analysis was of 10-week-old mice (*n* = 14 wild-type, 8 *rictor*^*+/−*^ and 7 L-RKO males; 12 wild-type, 6 *rictor*^*+/−*^ and 6 L-RKO females, * = *P* < 0.05). (D) Analysis of the phosphorylation of mTORC1 substrates in the livers of wild-type and L-RKO mice fasted overnight and then refed for 3 h. (E) Quantification of the phosphorylation of the mTORC1 substrates S6K1 T389, 4E-BP1 T37/S46, and 4E-BP1 S65, and the S6K1 substrate S6 S240/244 relative to each total protein in 10-week-old mice (*n* = 6 wild-type, 7 L-RKO males; 6 wild-type, 6 L-RKO females, * = *P* < 0.05).

In search of an explanation for the sexually dimorphic effect of Rictor depletion on longevity, we analyzed mTORC1 and mTORC2 signaling in male and female *rictor*^*+/−*^ and L-RKO mice (Fig. 3). We focused on the liver, as deletion of *Rictor* in this single tissue is sufficient to impact male lifespan. We observed a significant decrease in protein levels of RICTOR in both male and female *rictor*^*+/−*^ mice, as well as in male and female L-RKO mice (Fig. 3A–C). Depletion of RICTOR was similar in female and male mice, demonstrating that the sexually dimorphic effect of RICTOR depletion is not due to differential depletion of hepatic RICTOR.

mTORC2 directly phosphorylates two distinct sites on AKT T450 and S473 and has also been reported to regulate AKT protein levels (Sarbassov *et al*., 2005; Ikenoue *et al*., 2008; Hagiwara *et al*., 2012). Despite the decreased levels of RICTOR in *rictor*^*+/−*^ mice, we did not observe decreased phosphorylation of either mTORC2 site or a significant change in AKT protein levels (Fig. 3A–C). In contrast, we found a significant decrease in AKT protein expression in L-RKO mice, along with a dramatic decrease in phosphorylation of AKT T450 and S473 (Fig. 3B–C). Interestingly, we did not observe a significant effect of RICTOR depletion on the phosphorylation of AKT T308, demonstrating that phosphorylation of AKT T450 and S473 is not absolutely required for phosphorylation of this site in hepatic tissue.

We also considered the possibility that decreased levels of RICTOR could lead to increased mTORC1 activity, as we have previously shown that mTORC1 and mTORC2 can compete for limiting amounts of the mTOR protein kinase (Lamming *et al*., 2012), and chronic activation of mTORC1 would likely decrease longevity (Auricchio *et al*., 2012; Menon *et al*., 2012). Focusing again on the livers of the L-RKO mice, we examined the phosphorylation of the mTORC1 substrates S6K1 T389 and 4EBP-1 T37/S46 and S65, as well as S240/244 of ribosomal protein S6 (Fig. 3D). We found no significant differences in the phosphorylation of any of these residues in L-RKO mice compared to control mice.

### Male and female L-RKO mice are glucose intolerant

**Figure 4:**
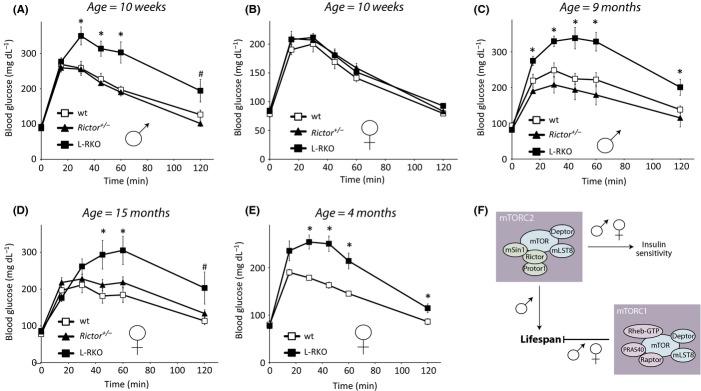
L-RKO mice, but not *rictor*^*+/−*^ mice, have impaired glucose tolerance. (A) Glucose tolerance is impaired in 10-week-old male L-RKO mice, but not in male *rictor*^*+/−*^ mice (*n* = 7 wt, 7 *rictor*^*+/−*^, 7 L-RKO). (B) Glucose tolerance test performed on 10-week-old female wt, *rictor*^*+/−*^ and L-RKO mice (*n* = 9 wt, 8 *rictor*^*+/−*^, 6 L-RKO). (C) Glucose tolerance is impaired in 9-month-old male L-RKO mice, but not *rictor*^*+/−*^ mice (*n* = 10 wt, 6 *rictor*^*+/−*^, 8 L-RKO). (D) Glucose tolerance is impaired in 15-month-old female L-RKO, but not *rictor*^*+/−*^ mice (*n* = 7 wt, 10 *rictor*^*+/−*^, 6 L-RKO). (E) Impaired glucose tolerance in female L-RKO mice at 4 months of age (*n* = 5 wt, 8 L-RKO). In all panels, *= *P* < 0.05, # = *P* < 0.06 L-RKO vs. wild-type, two-tailed *t*-test; error bars indicate standard error. (F) Model for the regulation of lifespan by mTORC1 and mTORC2.

We have previously demonstrated that deletion of *Rictor* specifically in the liver results in hepatic insulin resistance, as well as hyperglycemia and hyperinsulinemia, in young male L-RKO mice (Lamming *et al*., 2012). While the effect of insulin sensitivity on lifespan is complex – some insulin-resistant mice, such as mice lacking *IRS1*, are long lived (Taguchi *et al*., 2007; Selman *et al*., 2008, 2011) – many long-lived mouse models have increased insulin sensitivity, and diet-induced insulin resistance shortens mammalian lifespan (Kenyon, 2001). We examined glucose tolerance in young male and female *rictor*^*+/−*^ and L-RKO mice and were surprised to find that while 10-week-old male L-RKO mice were glucose intolerant, L-RKO female mice and *rictor*^*+/−*^ mice of both sexes had normal glucose tolerance (Fig. 4A,B). However, upon examining older L-RKO and *rictor*^*+/−*^ mice, we found that while *rictor*^*+/−*^ mice of both sexes continued to maintain wild-type glucose tolerance as they aged, both male and female L-RKO mice developed glucose intolerance (Fig. 4C,D). Further investigation revealed that L-RKO females develop glucose intolerance by four months of age (Fig. 4E). We also tested insulin sensitivity in aged male and female mice by means of an insulin tolerance test (Figure S3A, S3B). We found that aged female L-RKO mice were significantly more insulin resistant than aged-matched wild-type or *rictor*^*+/−*^ mice (Figure S3B).

## Discussion

Inhibition of mTOR signaling by rapamycin has been shown to significantly increase lifespan in mice and other organisms, but the specific role of mTORC1 and mTORC2 in this process has until now been unclear. While inhibition of mTORC1 clearly extends mammalian lifespan, the effect of mTORC2 inhibition on longevity has not been examined previously. In this study, we demonstrate that inhibition of mTORC2 signaling, either specifically in the liver or in the entire body, significantly decreases male but not female lifespan.

Herein, we used three different mouse models of Rictor depletion to examine the effect of decreased mTORC2 signaling on longevity. The commonality of these models is that all three – L-RKO, *rictor*^*+/−*^ and UbC-RKO mice – have a significant decrease in male lifespan. L-RKO and *rictor*^*+/−*^ female mice have a normal lifespan, despite comparable deletion of Rictor, while UbC-RKO female mice are visually indistinguishable from wild-type mice and are not obviously short lived. We also observed that a CR diet does not rescue the short lifespan of UbC-RKO male mice and that Rictor depletion may impair the effect of CR on glucose homeostasis and physical performance. The interpretation of this data is complicated by the short lifespan and deleterious phenotype of the UbC-RKO mice, but suggests that future studies on the role of mTORC2 in the response to CR may be worthwhile.

The pathologic and molecular mechanism by which RICTOR depletion shortens male lifespan remains obscure. We initially suspected that the sexually dimorphic effect of RICTOR depletion on lifespan was due to differential expression of RICTOR, but we found no effect of gender upon hepatic RICTOR depletion in either *rictor*^*+/−*^ or L-RKO mice (Fig. 3A–C). L-RKO mice of both genders have a significant decrease in the phosphorylation of the mTORC2 targets AKT T450 and S473, while displaying normal phosphorylation of AKT T308. Interestingly, the Hall and Soukas labs have recently found that expression of active AKT is sufficient to rescue the glucose tolerance and gluconeogenesis defect in L-RKO mice (Hagiwara *et al*., 2012; Yuan *et al*., 2012), suggesting that AKT activity is compromised in L-RKO mice despite the apparently normal phosphorylation of AKT T308. This may be due either to decreased total levels of AKT as we observe here or changes in activity mediated by AKT T450 or S473.

The lack of any defect in phosphorylation of the mTORC2 targets AKT T450 and S473 in *rictor*^*+/−*^ mice, combined with our data demonstrating that both male and female L-RKO mice develop glucose intolerance while *rictor*^*+/−*^ mice of both sexes remain glucose tolerant (Fig. 4), suggests that the sexually dimorphic effect of RICTOR depletion upon lifespan in L-RKO mice is likely not mediated by the activity of mTORC2 toward AKT in the liver, or by the development of hepatic insulin resistance. The delay in glucose intolerance of female L-RKO mice, which do not display glucose intolerance at 10 weeks of age (Fig. 4) despite similar decreases in mTORC2 signaling (Fig. 3), may be a function of the protective effects of estrogens, which have been shown to protect female mice against diet-induced glucose intolerance (Riant *et al*., 2009). Mice heterozygous for *Akt1* have an extended lifespan (Nojima *et al*., 2013), again suggesting that the impairment of male lifespan by RICTOR depletion may not be mediated by decreased AKT signaling.

We also do not observe altered hepatic mTORC1 activity following RICTOR depletion, in agreement with our recent proteomic and microarray analysis of L-RKO mice (Lamming *et al*., 2014). Physiological evidence also suggests that L-RKO mice do not have increased hepatic mTORC1 activity; while increased mTORC1 activity increases liver size in liver-specific *TSC1* knockout mice (Sengupta *et al*., 2010), L-RKO mice actually have smaller livers (Hagiwara *et al*., 2012). The molecular mechanism downstream of RICTOR/mTORC2 that mediates male lifespan is therefore unknown.

For now, the question of why RICTOR depletion results in decreased lifespan only in males remains unanswered. Identifying the mechanism underlying this effect will be a significant area of future study. Our work results provide evidence for a critical role of RICTOR, likely mediated by decreased mTORC2 signaling, in the health and survival of adult male mice (Fig. 4F). Our work has significant clinical relevance, as inhibition of mTORC2 has been suggested as a possible therapeutic approach for the treatment of certain cancers, and dual mTOR/PI3K inhibitors are being developed for clinical use. Our results suggest that prolonged use of such compounds may have detrimental effects in males.

Finally, rapamycin has attracted significant attention as a possible therapy for age-related diseases, but it was recently realized that prolonged treatment with rapamycin inhibits both mTORC1 and mTORC2 signaling. Treatment with rapamycin prolongs female lifespan by a greater percentage than male lifespan (Miller *et al*., 2013), and we hypothesize that this may be due in part to the male-specific detrimental effects of disrupted mTORC2 signaling. It will be interesting to determine whether other mouse models of extended longevity with greater positive effects on female lifespan likewise exhibit decreased mTORC2 signaling in males. Our results suggest that mTORC1-specific inhibitors may show greater efficacy than rapamycin in the extension of male lifespan.

## Experimental Procedures

### Materials

Antibodies to phospho-Akt S473 (4060), phospho-Akt T450 (9267), phospho-Akt T308 (9275), Akt (4691), phospho-p70 S6 kinase (9234), p70 S6 kinase (2708), phospho-S6 ribosomal protein (2215), S6 ribosomal protein (2217), p-4EBP1 S65 (9451), p-4EBP1 T37/46 (2855), total 4EBP1 (9452), β-tubulin (2146), and RICTOR (2140) were from Cell Signaling Technology. Protease and phosphatase inhibitor cocktail tablets were from Roche (11836153001 and 04906845001, respectively). Tamoxifen was purchased from VWR (IC15673891). Other chemicals were purchased from Sigma unless noted.

### Immunoblotting

Cells and tissue samples were lysed in cold RIPA buffer supplemented with phosphatase inhibitor and protease inhibitor cocktail tablets. Tissues were lysed in RIPA buffer using a FastPrep 24 (M.P. Biomedicals) and then centrifuged. Protein concentration was determined by Bradford (Pierce Biotechnology). 20 μg protein was separated by sodium dodecylsulpahte-polyacrylamide gel electrophoresis (SDS-PAGE) on 8%, 10%, or 16% resolving gels (Invitrogen, Life Technologies, Grand Island, NY, USA). Imaging was performed using a ge imagequant las 4000 imaging station. Quantification was performed by densitometry using imagej software, and loading was verified by blotting for tubulin or total Akt, as indicated.

### Animals and treatments

Rictor floxed mice were generated as described in (Guertin *et al*., 2009) and backcrossed to C57BL/6 at least 6 generations. Albumin-Cre mice on the C57BL/6 strain background were obtained from the Koch Institute Transgenic Facility. Mice heterozygous for *Rictor* were generated by crossing Rictor floxed mice with CMV-Cre mice obtained from the Jackson Laboratory (Strain Name: B6.C-Tg(CMV-cre)1Cgn/J, Stock Number: 006054) and crossing the progeny to C57BL/6 mice to remove the CMV-Cre transgene. Ubiquitin C-CreERT2 mice were obtained from the Jackson Laboratory (Strain Name: B6;129S-Tg(UBC-cre/ERT2)1Ejb/J, Stock Number: 007001). Tamoxifen was suspended in sunflower seed oil at a concentration of 10 mg mL^−1^, and 200 μL per 25 g of body weight was injected intraperitoneally once daily for 7 days. Control animals received an equal volume of the tamoxifen suspension, but did not express the CreERT2 fusion protein. Following tamoxifen treatment, mice for the CR diet study were allowed to recover for 1 week and were then placed on an *ad-libitum* diet or CR (80% of AL food intake) diet. After one additional week, CR animals were further restricted to 60% of AL food intake. Glucose and insulin tolerance tests were performed by fasting the mice overnight for 16 h and then injecting either glucose (2 g kg^−1^) or insulin (0.75 U kg^−1^) intraperitoneally. Glucose measurements were performed using a Bayer Contour blood glucose meter and test strips.

### Lifespans and statistics

Lifespans were performed using the L-RKO, *rictor*^*+/−*^ and UbC-RKO mouse strains. The genetic background was C57BL/6, and wild-type littermates were used for the control groups. Mice were housed with no more than five mice per cage, except for the UbC-RKO CR diet study, in which there was no more than five mice per cage. Genotyping was performed as previously described (Guertin *et al*., 2006; Lamming *et al*., 2012). For the L-RKO and *rictor*^*+/−*^ mouse lifespan studies, the survival of 155 mice was analyzed as follows: Females (39 wild-type, 21 *rictor*^*+/−*^, 33 L-RKO); Males (30 wild-type, 14 *rictor*^*+/−*^, 18 L-RKO). For the UbC-RKO CR lifespan study, the survival of 52 mice was analyzed as follows: Males (11 wild-type AL, 9 wild-type CR, 15 UbC-RKO AL, 13 UbC-RKO CR). For the UbC-RKO lifespan study starting at 9 months of age, the survival of 25 mice was analyzed as follows: Males (10 wild-type, 15 UbC-RKO). Mice were fed a diet of either RMH 3000 chow diet (Prolab), or for the CR study, NIH31, in MIT’s specific pathogen-free facility, with minimal disease status during the lifespan study. Survival was calculated using the date each mouse was found dead or was determined to be moribund by veterinary technicians. Criteria for euthanasia included a low body condition score (BC < 2, or less than 2- in the case of CR mice), visible tumors > 1 cm in diameter, severe dermatitis penetrating to the fascia, and inability to feed or drink. Survival statistics were calculated using prism (graphpad Software).

## Supporting Information

**Fig. S1** Rotarod performance of wild-type, *rictor*^*+/−*^ and L-RKO mice.

**Fig. S2** Depletion of RICTOR blunts the beneficial effects of a CR diet.

**Fig. S3** Insulin tolerance test on mice depleted for RICTOR.

**Table S1** Statistical analysis of the lifespans shown in Figure 1, 2, and S2.

**Table S2** Raw lifespan data for Figure 1, 2, and S2.

**Table S3** Analysis of causes of death for the lifespans in Figure 1.
