# Supplementary figures and images for: Depletion of Rictor, an essential protein component of mTORC2, decreases male lifespan
Source: Aging Cell. 2014 Jul 25;13(5):911–7. doi: 10.1111/acel.12256 (PMC4172536; doi:10.1111/acel.12256)

Figure S1

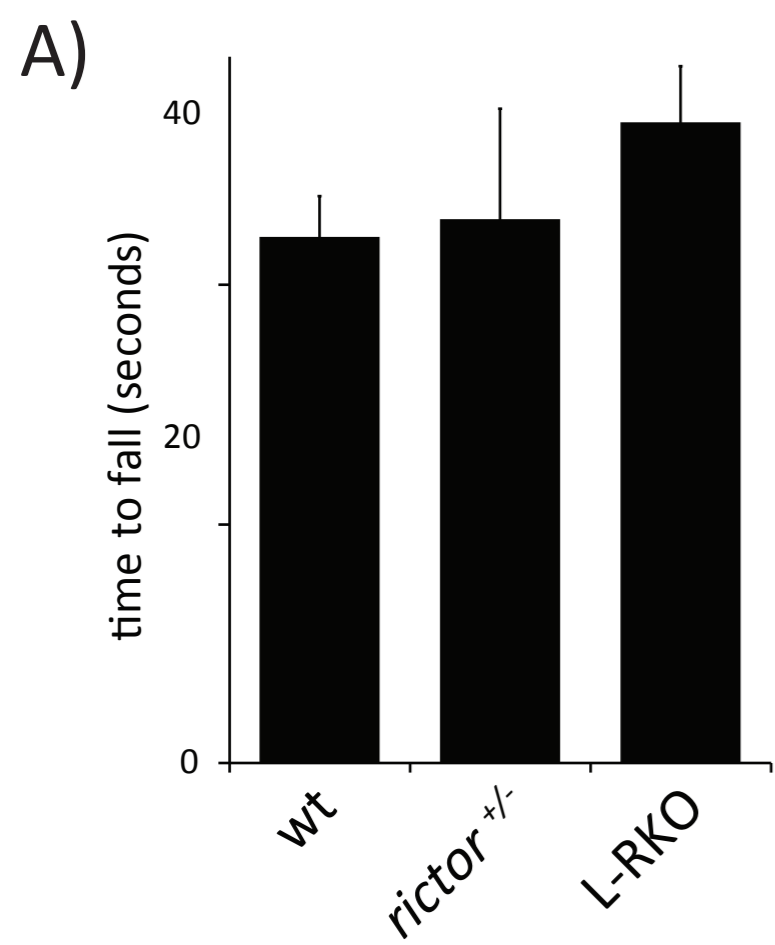

Supplement: Supplementary file 1 — Fig. S1 Rotarod performance of wild-type, rictor+/− and L-RKO mice. [file acel0013-0911-sd1.pdf]

Figure S2

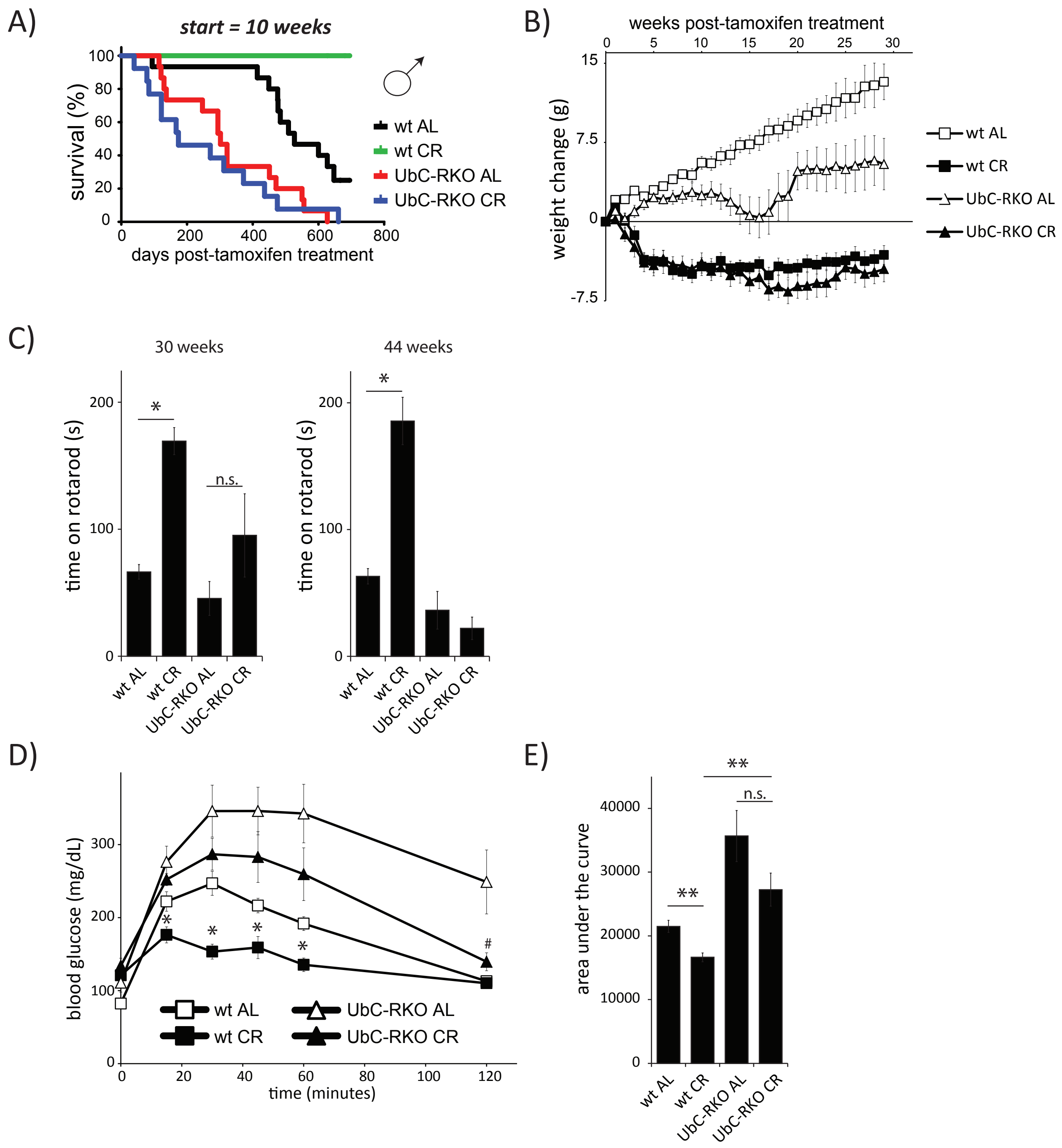

Supplement: Supplementary file 2 — Fig. S2 Depletion of RICTOR blunts the beneficial effects of a CR diet. [file acel0013-0911-sd2.pdf]

Figure S3

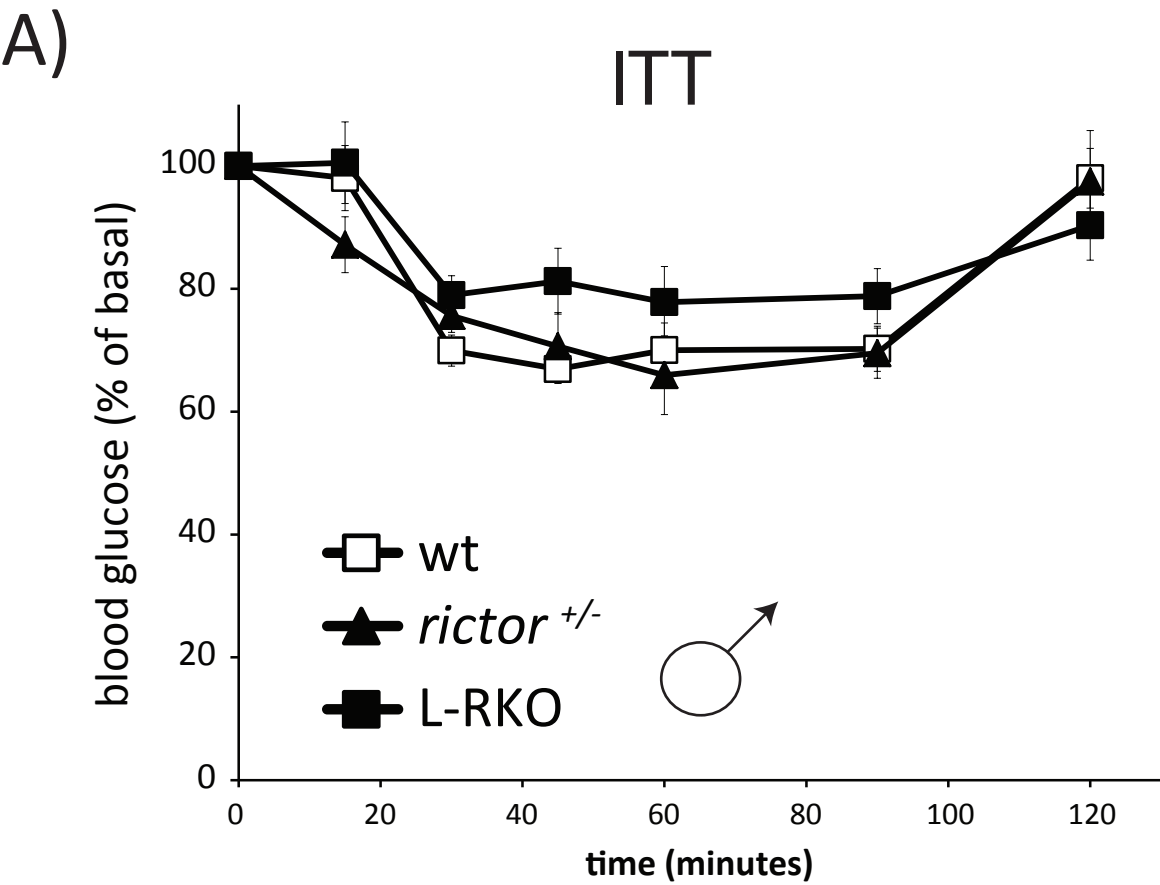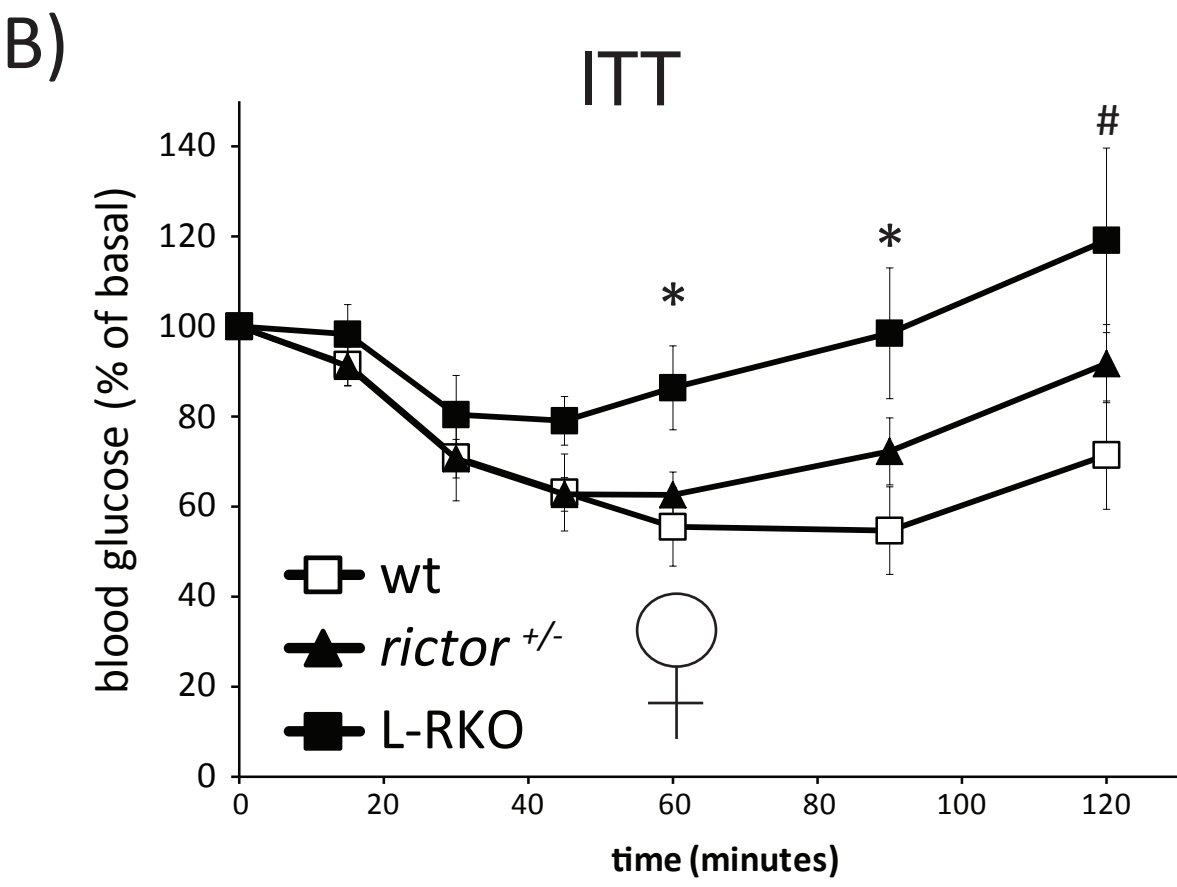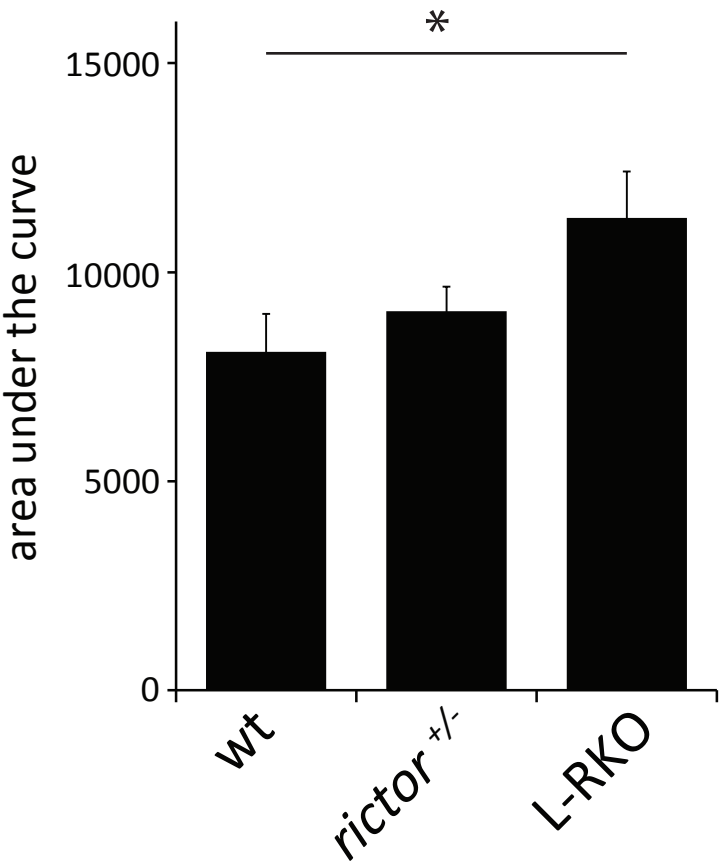

Supplement: Supplementary file 3 — Fig. S3 Insulin tolerance test on mice depleted for RICTOR. [file acel0013-0911-sd3.pdf]
