# Supplementary material for: Depletion of Rictor, an essential protein component of mTORC2, decreases male lifespan
Source: Aging Cell. 2014 Jul 25;13(5):911–7. doi: 10.1111/acel.12256 (PMC4172536; doi:10.1111/acel.12256)
Supplement: Supplementary file 4 — Table S1 Statistical analysis of the lifespans shown in Figure 1, 2, and S2. [file acel0013-0911-sd4.pdf]

Supplemental Table 1

Figure 1

Males		Mean	Median	P value, Log-rank test vs. wt
	wt	673	707	
	L-RKO	511	502	0.0016
	rictor	474	428	0.0002

Females		Mean	Median	P value, Log-rank test vs. wt
	wt	692	734	
	L-RKO	694	734	0.913
	rictor	750	785	0.127

Figure 2

Fig. 2A		Mean	Median	P value, Log-rank test vs. wt
	wt	---	527	
	UbC-RKO	329.3	302	0.0016

Fig. 2B		Mean	Median	P value, Log-rank test vs. wt
	wt	---	235	
	UbC-RKO	---	169	0.0013

Figure S2

Fig. S2A		Mean	Median	P value, Log-rank test vs. wt	vs. wt CR	vs. UbC-RKO AL
	wt AL	---	527		0.0013	0.0016
	wt CR	---	---	0.0013		< 0.0001
	UbC-RKO AL	329.3	302	0.0016	< 0.0001	
	UbC-RKO CR	254.8	174	0.0004	< 0.0001	0.5356
