# Supplementary material for: Depletion of Rictor, an essential protein component of mTORC2, decreases male lifespan
Source: Aging Cell. 2014 Jul 25;13(5):911–7. doi: 10.1111/acel.12256 (PMC4172536; doi:10.1111/acel.12256)
Supplement: Supplementary file 5 — Table S2 Raw lifespan data for Figure 1, 2, and S2. [file acel0013-0911-sd5.pdf]

Supplemental Table 2

Figure 1

Males

wt	L-RKO	*rictor* $^{+/-}$
297	322	340
347	325	363
354	341	364
404	345	374
464	393	388
491	397	409
499	400	417
522	477	438
635	489	460
654	515	483
657	534	503
662	591	647
662	599	684
662	624	769
686	638	
728	652	
732	656	
751	900	
753		
764		
769		
774		
782		
795		
826		
857		
894		
899		
900		
963		

Females

wt	L-RKO	*rictor* $^{+/-}$
165	449	448
269	462	458
312	467	549
432	468	633
541	490	645
565	512	661
584	516	676
590	612	684
616	615	737
629	628	750

Figure 2A

Males

wt	UbC-RKO
94	115
414	121
449	130
476	137
477	247
484	294
509	294
527	302
600	322
627	322
627*	451
647	471
666*	550
666*	556
694*	627

* = alive at conclusion of experiment

Figure 2B

Males

wt	UbC-RKO
217	49
235	105
253*	126
253*	135
279*	149
279*	153
279*	160
279*	169
306*	170
335	180
	248
	253*
	253*
	279*
	306*

* = alive at conclusion of experiment

631	629	785
633	632	802
654	647	813
667	658	836
675	674	846
680	687	854
694	734	865
703	747	871
720	749	909
734	753	936
736	756	991
737	756	
758	765	
759	767	
767	785	
773	808	
773	812	
782	845	
783	864	
810	875	
822	879	
837	903	
856	969	
867		
873		
879		
892		
896		
909		

Figure S2A * = alive at conclusion of experiment

Males

wt AL	wt CR	UbC-RKO AL	UbC-RKO CR
94	627*	115	39
414	627*	121	78
449	627*	130	84
476	627*	137	122
477	680*	247	122
484	680*	294	167
509	680*	294	174
527	680*	302	271
600	694*	322	312
627		322	372
627*		451	436
647		471	475
666*		550	661
666*		556	
694*		627	
