# Supplementary material for: Depletion of Rictor, an essential protein component of mTORC2, decreases male lifespan
Source: Aging Cell. 2014 Jul 25;13(5):911–7. doi: 10.1111/acel.12256 (PMC4172536; doi:10.1111/acel.12256)
Supplement: Supplementary file 6 — Table S3 Analysis of causes of death for the lifespans in Figure 1. [file acel0013-0911-sd6.pdf]

Supplemental Table 3

Males	% of mice observed with cancer at death	% of mice examined	number of mice examined
wt	54.2	80.0	24 of 30
rictor+/-	9.1	78.6	11 of 14
L-RKO	29.4	94.4	17 of 18

1 *rictor* $^{+/-}$ male was euthanized for head tilt, 1 *rictor* $^{+/-}$ male euthanized for a deep abscess, and 2 L-RKO males were euthanized due to difficulty breathing

Females	% of mice observed with cancer at death	% of mice examined	number of mice examined
wt	43.8	82.1	32 of 39
rictor+/-	31.6	90.5	19 of 21
L-RKO	28.1	97.0	32 of 33

4 wt females, 1 *rictor* $^{+/-}$ female and 3 L-RKO females were euthanized for ulcerations and dermatitis
